# New Medical School of Virginia

**Published:** 1838

**Authors:** 


Art. VII.—New Medical School in Virginia.

Another school for medical instruction, has been recently, organized in the capital of the "Old Dominion," under the title of the *Medical College of Richmond.* Its warrant is derived from Hampden-Sydney College. The following gentlemen compose its Faculty:—

TH. JOHNSON, M. D. (formerly professor of Aanatomy in the University of Virginia,) *Professor of Anatomy and Physiology.*

JOHN CULLEN, M. D. *Professor of Theory and Practice of Medicine.*

L.W. CHAMBERLAYNE, M. D. *Professor of Materia Medica and Therapeutics.*

R. L. BOHANNAN, M. D. *Professor of Obstetrics and Diseases of Women and Children.*

AUG. L. WARNER, M. D. (late professor of Anatomy and Surgery in the University of Virginia) *Professor of Surgery.*

SOCRATES MAUPIN, M. D., *Professor of Chemistry and Pharmacy.*

The session is to be five months long, which would seem to presage a more elaborate and extended course of study, than can be prosecuted in most of our existing schools. Such, however, will not be the fact, for another innovation is, that the pupil may become a candidate for a degree, at the end of one session. But why require an attendance for even one session, or that the candidate shall, as they publish, have studied for two years with a private preceptor? Do the Faculty of the Richmond school really believe, that two years of private tuition, and one course of lectures, are sufficient to qualify young men for the doctorate? If they do, we feel quite assured they are mistaken. If *any* time or course of study be prescribed, it should be made as long, as a sound judgment shall pronounce to be necessary, for the majority of our young men. Our schools should insist on *such* a routine, in connexion with an examination, to guard against the graduation of unworthy candidates. They will thus have a double guaranty.

They who rely on the examination alone, are more consistent than those who prescribe a time, but make it only a part of what it should be. We are in favor of examinations as a test, but still prefer to connect them with a specified course of studies. We cannot perceive, in what way our students are injured, by compelling them to do what we know must be done, before they can undergo good examinations. We hope the respectable founders of this school will retrace their steps, and fall into the track, which, in this matter, we consider the true path. D.

